# Estimating retention in HIV care accounting for patient transfers: A national laboratory cohort study in South Africa

**DOI:** 10.1371/journal.pmed.1002589

**Published:** 2018-06-11

**Authors:** Matthew P. Fox, Jacob Bor, Alana T. Brennan, William B. MacLeod, Mhairi Maskew, Wendy S. Stevens, Sergio Carmona

**Affiliations:** 1 Department of Epidemiology, Boston University School of Public Health, Boston, Massachusetts, United States of America; 2 Department of Global Health, Boston University School of Public Health, Boston, Massachusetts, United States of America; 3 Health Economics and Epidemiology Research Office, Department of Internal Medicine, School of Clinical Medicine, Faculty of Health Sciences, University of the Witwatersrand, Johannesburg, South Africa; 4 National Health Laboratory Service, Johannesburg, South Africa; 5 Department of Molecular Medicine and Haematology, University of the Witwatersrand, Johannesburg, South Africa; University of Southampton, UNITED KINGDOM

## Abstract

**Background:**

Systematic reviews have described high rates of attrition in patients with HIV receiving antiretroviral therapy (ART). However, migration and clinical transfer may lead to an overestimation of attrition (death and loss to follow-up). Using a newly linked national laboratory database in South Africa, we assessed national retention in South Africa’s national HIV program.

**Methods and findings:**

Patients receiving care in South Africa’s national HIV program are monitored through regular CD4 count and viral load testing. South Africa’s National Health Laboratory Service has maintained a database of all public-sector CD4 count and viral load results since 2004. We linked individual laboratory results to patients using probabilistic matching techniques, creating a national HIV cohort. Validation of our approach in comparison to a manually matched dataset showed 9.0% undermatching and 9.5% overmatching. We analyzed data on patients initiating ART in the public sector from April 1, 2004, to December 31, 2006, when ART initiation could be determined based on first viral load among those whose treatment followed guidelines. Attrition occurred on the date of a patient’s last observed laboratory measure, allowing patients to exit and reenter care prior to that date. All patients had 6 potential years of follow-up, with an additional 2 years to have a final laboratory measurement to be retained at 6 years. Data were censored at December 31, 2012. We assessed (a) national retention including all laboratory tests regardless of testing facility and (b) initiating facility retention, where laboratory tests at other facilities were ignored. We followed 55,836 patients initiating ART between 2004 and 2006. At ART initiation, median age was 36 years (IQR: 30–43), median CD4 count was 150 cells/mm^3^ (IQR: 81–230), and 66.7% were female. Six-year initiating clinic retention was 29.1% (95% CI: 28.7%–29.5%). After allowing for transfers, national 6-year retention was 63.3% (95% CI: 62.9%–63.7%). Results differed little when tightening or relaxing matching procedures. We found strong differences in retention by province, ranging from 74.2% (95% CI: 73.2%–75.2%) in Western Cape to 52.2% (95% CI: 50.6%–53.7%) in Mpumalanga at 6 years. National attrition was higher among patients initiating at lower CD4 counts and higher viral loads, and among patients initiating ART at larger facilities. The study’s main limitation is lack of perfect cohort matching, which may lead to over- or underestimation of retention. We also did not have data from KwaZulu-Natal province prior to 2010.

**Conclusions:**

In this study, HIV care retention was substantially higher when viewed from a national perspective than from a facility perspective. Our results suggest that traditional clinical cohorts underestimate retention.

## Abstract 2

In a large observational study, Matthew Fox and colleagues investigate retention in HIV care in South Africa allowing for patient transfers between clinics.

## Author summary

**Why was this study done?:**

- Since the large-scale rollout of HIV treatment in resource-limited settings, evidence suggests that retention in HIV treatment programs (i.e., patients being alive and in care) has been suboptimal.
- However, to date, most analyses have been from the perspective of individual cohorts where researchers do not have the ability to trace patients who move from one HIV treatment site to another, something we suspect is common.
- Failure to account for patient movement between clinics (sometimes referred to as “silent transfer”) can make estimates of retention in care seem worse than they really are, and this has implications for planning and where to target interventions to reduce losses from HIV programs.

**What did the researchers do and find?:**

- We took advantage of the fact that South Africa has a national provider of laboratory investigations, the National Health Laboratory Service (NHLS), that has maintained results on most patients in South Africa since the beginning of the treatment program in 2004 (with the exception of KwaZulu-Natal province).
- We used probabilistic matching techniques to turn these data into a longitudinal cohort that could be used to determine when patients were lost to follow-up and when they moved between clinics.
- This allowed us to compare retention in care from the perspective of the clinic (the perspective most often reported) and from the national perspective, which accounts for movement between sites.
- We found that from the clinic perspective, among patients who started HIV treatment according to national guidelines between 2004 and 2006, 29.1% were still in care 6 years later.
- However, when looking from a national perspective, which accounted for movement between clinics, retention was estimated to be 63.3% at 6 years.

**What do these findings mean?:**

- Our findings suggest that there is substantial movement between HIV clinics in South Africa, and that ignoring these movements has the potential to strongly underestimate retention in HIV care.
- Future efforts to see if these trends continue in later cohorts will be important to developing effective interventions aimed at reducing loss from HIV care.

## Introduction

In 2015, the World Health Organization (WHO) recommended removing CD4 count thresholds for HIV treatment eligibility [1] based on clinical trial evidence showing benefits to patients [2,3] and reduced transmission to uninfected partners [4]. It is hoped this recommendation will increase the number of patients on antiretroviral therapy (ART) and reduce new infections [5–9]. However, WHO specifically noted that the expected gains of such an approach could only be achieved if improvements were made in retaining patients on ART.

To date, estimates of retention worldwide in HIV treatment programs have varied widely. In a 2015 review, we found that global retention was 74% at 24 months and 60% at 60 months [10]. Results were similar when limited to sub-Saharan Africa and to children [11]. The existing retention literature is based on clinical research cohorts [12–15] and thus has limitations. First, estimates typically come from large, well-resourced clinics, which may have different retention rates than small and under-resourced clinics. Second, these cohorts cannot describe the diversity of experiences within countries as they typically come from urban areas. Third, most capture only clinic retention and do not track patients lost to follow-up. Patients who move to a new clinic without informing their prior clinic [16], so-called silent transfers [17–21], are considered lost even though they may be in care. Taken together, it is difficult to know whether current retention estimates over- or underestimate retention, information critical to tracking success towards the Joint United Nations Programme on HIV/AIDS (UNAIDS) 90-90-90 targets [22].

In contrast, the numbers estimated to be on ART in cross-sectional analyses suggest that a higher proportion of patients who have started ART are still in care [23]. It is unknown whether this apparent contradiction reflects bias in cohort retention estimates or overreporting by health facilities. If ART retention is low, the population health benefits of ART scale-up [24] could be transitory. If retention is higher, the benefits documented thus far could reflect long-lasting improvements in population health.

The impact of patient movement may potentially be a large source of bias in retention estimates in countries like South Africa where in-country migration is common [25]. Additionally, as ART scale-up continues and patients have more choices for where to access treatment, patients may seek care at clinics that are more convenient, offer more anonymity, or offer higher perceived quality. Recent data from Gauteng, South Africa, have shown that movement between clinics is also common among postpartum women [25]. If patients do link to a new site, this is a positive outcome, but it is difficult to track patients across sites when no formal transfer is requested, and national retention estimates are likely impacted.

Until now, lack of a national, integrated clinical ART database in South Africa has made it difficult to determine if retention outcomes observed in research cohorts are nationally representative, and we have not been able to correct for silent transfers (though approaches have been proposed [16,26–28]). Using a national South African HIV cohort created via novel linkage of routine laboratory monitoring data for the entire national HIV program, we assessed both clinic-level and national retention accounting for movement across facilities.

## Methods

### Overview of analytic approach

We used probabilistic matching to link individual laboratory results to transform South Africa’s national laboratory database into a national longitudinal HIV cohort [29,30]. The cohort provided an opportunity to estimate national retention since the inception of the public-sector treatment program in 2004 and to evaluate the impact of patient movement on retention. During the study period, patients were monitored through 6-monthly CD4 counts and viral loads to determine ART eligibility and monitor treatment. While the database does not contain patient visits or ART initiation dates [31], we used lab monitoring as evidence of retention and lab monitoring protocols to impute ART initiation (details below). Finally, we described retention from ART initiation in relation to key factors that might predict retention.

### Data source and study population

The National Health Laboratory Service (NHLS) provides nearly all diagnostic pathology services for South Africa’s public-sector health system, including all CD4 count and viral load tests conducted in public-sector facilities since ART rollout in 2004 (KwaZulu-Natal joined NHLS in 2010 and is excluded from analyses). All laboratory test results are stored at the NHLS Corporate Data Warehouse. Patients do not pay for treatment or for testing to be done.

NHLS data include patient name, birth date, sex, facility, test dates, and results. Because the database has no unique patient identifier, we created a probabilistic record-linkage algorithm, using a modified version of the Fellegi–Sunter method [32,33], in which comparisons between labs results were made on first name, last name (surname), birth date, sex, facility, and province. We note that patients could move between facilities and provinces and still be a match. Because the linkage was probabilistic, lab results occurring in the same province were considered more likely to belong to the same patient than lab results occurring in different provinces. However, these results from different provinces could be attributed to the same patient if there was sufficiently high agreement on other identifiers, e.g., first name, last name, date of birth, and sex. Matching elements were weighted based on frequency of responses (rare name matches had more weight than common name matches). A weighted average over component scores was created, with weights optimized using manually matched training data. As typological errors are common, we used the Jaro–Winkler [33,34] approach to string comparisons (first name, last name) and integrated these into the score [35,36]. We used deterministic record linkage to search for name inversions and linkage on middle/maiden names, and to match on a list of >16,000 nicknames, translated names, and common misspellings. We applied graph-based network analysis approaches to identify and break up improbable clusters.

We validated our approach against a manually matched quasi-gold standard developed by manually coding a random sample of >59,000 potential matches. All of these potential matches were validated by having a team of research assistants adjudicate if the match was indeed a true match. Our algorithm performed well in terms of avoiding overmatching (i.e., linkage of records that are not true matches, which creates the impression that patients were in care longer than they were) and undermatching (i.e., failure to link true matches, which creates the impression of 2 patients with shorter retention). We found a sensitivity of 91.0% (i.e., 9.0% undermatching) and positive predictive value of 90.5% (i.e., 9.5% overmatching). We additionally used our algorithm to identify the subset of 55,836 patients who were linked with high confidence, i.e., those who neither lost nor gained records as the threshold to define a match was varied. Because overmatching could lead to falsely identifying transfers across facilities, we restricted the study sample to these high-quality matches (84.8% of all patients and 74.8% of all lab results) for our primary analysis. In robustness checks, we included all 72,256 patients and completed other sensitivity analyses using less restrictive criteria (described below) that included up to 118,720 patients.

We included patients initiating ART during the period April 1, 2004–December 31, 2006, when viral load testing was recommended at ART staging [37]. While a viral load at initiation was recommended in treatment guidelines, implementation of these guidelines varied by site, and thus our results apply only to those patients who we observed as having a viral load test conducted at treatment initiation. Patients whose first viral load was suppressed (<1,000 copies/ml) were excluded as they were assumed to have transferred in from the private sector. We excluded patients from sites with <10 patients starting ART during 2004–2006, as these likely were not routine HIV clinics. As patients could get a baseline viral load measurement but not initiate ART, for our primary analysis we included patients with 2 viral load measures (>30 days apart) to ensure that patients initiated treatment. While this improved our ability to ensure patients were on treatment, it meant we would miss some early mortality in this analysis as we would code such attrition as attrition of a patient not yet on treatment. In a sensitivity analysis, we included all 118,720 patients with a first viral load, noting that this included a mix of patients who did and did not initiate ART. We conducted another sensitivity analysis with stricter inclusion criteria, including only those with 2 viral loads and who had an alanine transaminase (ALT) or hemoglobin test (tests used in an ART workup) within ±90 days of the first viral load (*n =* 19,415). We defined ART initiation to have occurred at the date of the first viral load.

### Retention and transfer

Attrition (defined as 1 minus the proportion of patients who were retained) included death and loss to follow-up as we cannot distinguish between the 2 events with our data. As clinic visit dates are not available, we defined retention based on laboratory monitoring. During the study period, there were some changes in monitoring protocols; however, the longest a patient should have gone without monitoring is 1 year.

We defined attrition retrospectively, with attrition occurring on the patient’s last observed laboratory date, allowing patients to exit and reenter care prior to that date. Patients contributed person-time from ART initiation (first viral load) until the earlier of attrition or December 31, 2012. All patients had 6 potential follow-up years plus an additional 2 years (to December 31, 2014) to have a laboratory measurement to determine retention at end of follow-up (8 years total). Patients with a lab test in those additional 2 years were considered retained at 6 years; otherwise, they were considered lost and censored at their last lab date. Two years was used to allow patients a grace period of 1 year after the maximum period a patient should have had between viral loads, which was 1 year. We censored follow-up at the end of 2012 as some facility identifiers in the NHLS dataset changed in 2015, making it difficult to identify retention at the same clinic. For this analysis, patients contributed person-time up to the time of their last viral load even if they missed viral load tests in between.

Numerous definitions of retention have been proposed [38–41]. We defined retention from 2 perspectives: retention at the initiating facility (clinic perspective) and retention at any clinic within the public-sector health system (national perspective). From the clinic perspective, we defined retention based on lab tests only at the initiating clinic, while from the national perspective we included all labs regardless of location. We compared the national perspective to the clinic perspective of most published cohorts, but used the national perspective for identifying attrition predictors. We note that this definition of retention is focused on whether a person is in care at the end of follow-up and not whether or not the person has been continually in care since the time that they initiated treatment. Patients, in this definition, can enter care, leave care for a period of time, and reenter care and still be considered retained. In another sensitivity analysis to address the issue of cycling in and out of care, we defined retention using a prospective definition in which attrition occurred the first time a patient went 2 years without a laboratory measure (prospective definition). This approach describes the first time a person becomes lost, in contrast to our primary, retrospective, definition, which describes whether a patient is in care at the end of follow-up despite gaps. Here we refer to patients retained according to the prospective definition as continually retained, to indicate that they met the retention definition at each time point.

We defined transfer between clinics as a patient having a laboratory test at a clinic other than their initial clinic. We defined the transfer date as the last date the patient was at their initiating clinic. We cannot distinguish between formal transfer (where a patient notifies the clinic) and silent transfer (where the patient switches clinics without informing the initiating clinic). For the analysis of transfers, we considered only the first movement between clinics and did not consider further movements to a third clinic or back to the initiating clinic. Patients who made multiple moves were still considered retained in the retention analysis as long as they continued to meet the retention definition. CD4 count at ART initiation was defined as the value closest to initiation that was between 12 months prior to and 3 months after a patient’s date of first viral load.

### Statistical methods

We did not do any sample size calculations but included all patients in the national cohort who met the inclusion criteria. We described retention using Kaplan–Meier curves of time since ART initiation from both the clinic and the national perspective. We stratified analyses by sex, age, CD4 count and viral load at ART initiation, clinic size (total number of patients who had initiated ART by the end of 2006, divided into quintiles), and province. We assessed predictors of retention using Cox proportional hazards regression. Finally, we looked at movement between provinces by classifying patients by whether they moved to facilities outside their initiating province and, if not, by any movement between facilities within their initiating province. We did not impute missing data but do report the numbers missing from analyses. We did not have a pre-established analysis plan or published protocol.

The study was approved by the Human Research Ethics Committee of the University of the Witwatersrand and the Boston University Institutional Review Board for use of de-identified data with a waiver of consent.

## Results

The NHLS cohort contained 55,836 people who initiated ART in South Africa between 2004 and 2006 (Table 1). The population was predominately female (66.7%) with a median age of 36 years (IQR: 30–43) at ART initiation. Gauteng province contributed the largest share of patients, at 29.0%, while Northern Cape had the smallest, at 3.1% (KwaZulu-Natal was excluded from all analyses). Median CD4 count at ART initiation (closest to first viral load but no more than 12 months before and more than 90 days after) was 150 cells/mm^3^ (IQR: 81–230).

**Table 1:** Characteristics of patients in South Africa’s national HIV cohort stratified by province (*n =* 55,836).

Characteristic	Measure	Eastern Cape (*n =* 11,118, 19.9%)	Free State (*n =* 3,529, 6.3%)	Gauteng (*n =* 16,269, 29.1%)	Limpopo (*n =* 3,546, 6.3%)	Mpumalanga (*n =* 3,876, 6.9%)	Northern Cape (*n =* 1,754, 3.1%)	North West (*n =* 8,713, 15.7%)	Western Cape (*n =* 7,031, 12.7%)	Total (*n =* 55,836)
Female	*n* (%)	7,598 (70.0)	2,316 (66.6)	10,380 (64.5)	2,440 (69.8)	2,624 (68.2)	1,165 (67.0)	5,548 (64.4)	4,662 (67.0)	36,733 (66.7)
Age (years) at first viral load	Median (IQR)	35 (29–43)	37 (31–44)	37 (31–44)	38 (32–46)	37 (31–45)	35 (29–43)	36 (31–43)	34 (29–40)	36 (30–43)
CD4 count at first viral load (cells/mm^3^)*	Median (IQR)	172 (99–275)	133 (71–185)	133 (68–197)	162 (90–276)	150 (81–236)	147 (78–230)	145 (74–229)	163 (93–250)	150 (81–230)
	Missing *n* (%)	946 (8.5)	656 (18.6)	2,814 (17.3)	397 (11.2)	958 (24.7)	113 (6.4)	900 (10.3)	963 (13.7)	7,747 (13.4)
First viral load (copies/1,000 ml)	Median (IQR)	88 (23–310)	80 (24–240)	97.5 (26–310)	106 (28–290)	85.6 (23–290)	84 (21–280)	90 (25–280)	89 (25–280)	91 (24–300)
Retained	*n* (%)	7,290 (65.6)	2,303 (65.3)	9,782 (60.1)	2,003 (56.5)	2,022 (53.2)	1,134 (64.7)	5,601 (64.3)	5,218 (74.2)	35,353 (63.3)
Time between viral load measures (days)	Median (IQR)	215 (170–353)	194 (168–300)	203 (168–336)	196 (170–290)	217 (178–364)	195 (169–292)	195 (168–273)	185 (164–237)	198 (168–309)
Median number of lab tests per year**	Median (IQR)	4 (2–6)	4 (2–6)	3 (2–6)	4 (2–6)	3 (2–5)	4 (2–6)	4 (2–6)	4 (2–6)	4 (2–6)

*CD4 count at first viral load was defined as the value closest to the date of the first viral load that was between 12 months prior to and 3 months after the date of the first viral load.

**Any lab test type (alanine transaminase, hemoglobin, viral load, or CD4 count).

**Fig 1:**
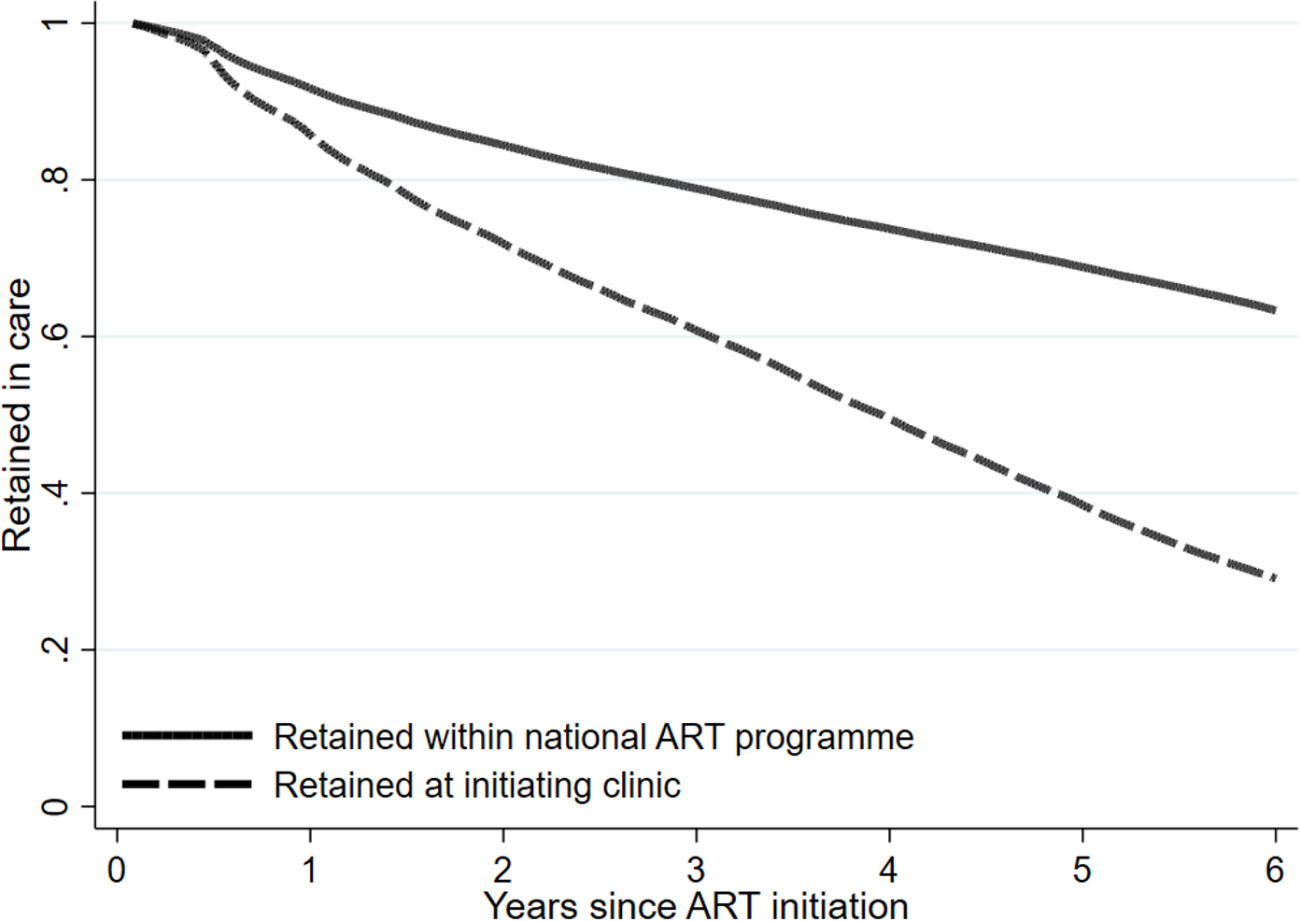
Effect of patient transfer on retention estimates overall in South Africa among 55,836 patients initiating ART in 2004–2006, with attrition defined as retained in care on December 31, 2012.

Six-year retention for the national cohort is shown in Fig 1, with the solid line reflecting retention from a national perspective and the dashed line that from the clinic perspective. From the clinic perspective, attrition was steady and strong over the entire 6 years of follow-up. From the national perspective, attrition was also consistent over the 6 years. Clinic retention at 6 years, assuming anyone who met the attrition definition at the site was truly lost, approached 29.1% (95% CI: 28.7%–29.5%). This differs from national retention, in which 63.3% of patients (95% CI: 62.9%–63.7%) were retained, a 34.2 percentage point difference from the clinic perspective.

S1 Appendix shows a sensitivity analysis in which we conducted the same analysis but using a prospective retention definition in which any patient who experienced a gap of 24 months without laboratory monitoring was considered to have reached attrition even if they returned to care later. Here, when using a “continually retained” definition of retention, we see less of a discrepancy between the 2 perspectives, but with the national retention curve still substantially higher. We found that for patients who left care at 1 clinic and reappeared at another, the median (IQR) time between laboratory tests was 3.8 months (1.2–7.4). The median number of transfers per person was 1 (IQR: 0–2). The results changed little when we relaxed our matching procedures (S2 Appendix), where we saw a 66% national and 29% clinic retention rate at 6 years. In addition, when we tightened the inclusion criteria by limiting the analysis to those with 2 viral loads plus a hemoglobin or ALT measurement within 90 days before or after their first viral load (to ensure a high likelihood of ART initiation), the results also changed very little (62% versus 25% retention) (S3 Appendix). The difference between the clinic and national perspectives also changed little when we included all patients with at least 1 viral load, but the overall retention rates dropped substantially as this included patients who had a viral load but never returned for additional monitoring (S4 Appendix). In addition, this drop in the retention rates likely also reflects a mixing in of persons who never initiated treatment even though they did have a first viral load. We also repeated the analysis limited to those with a CD4 count at ART initiation of <200 cells/mm^3^ (i.e., those who would have been eligible for ART initiation based only on their CD4 count) and saw little change in the results (S5 Appendix). The true results thus likely lie somewhere between our primary results and the sensitivity analysis results.

**Fig 2:**
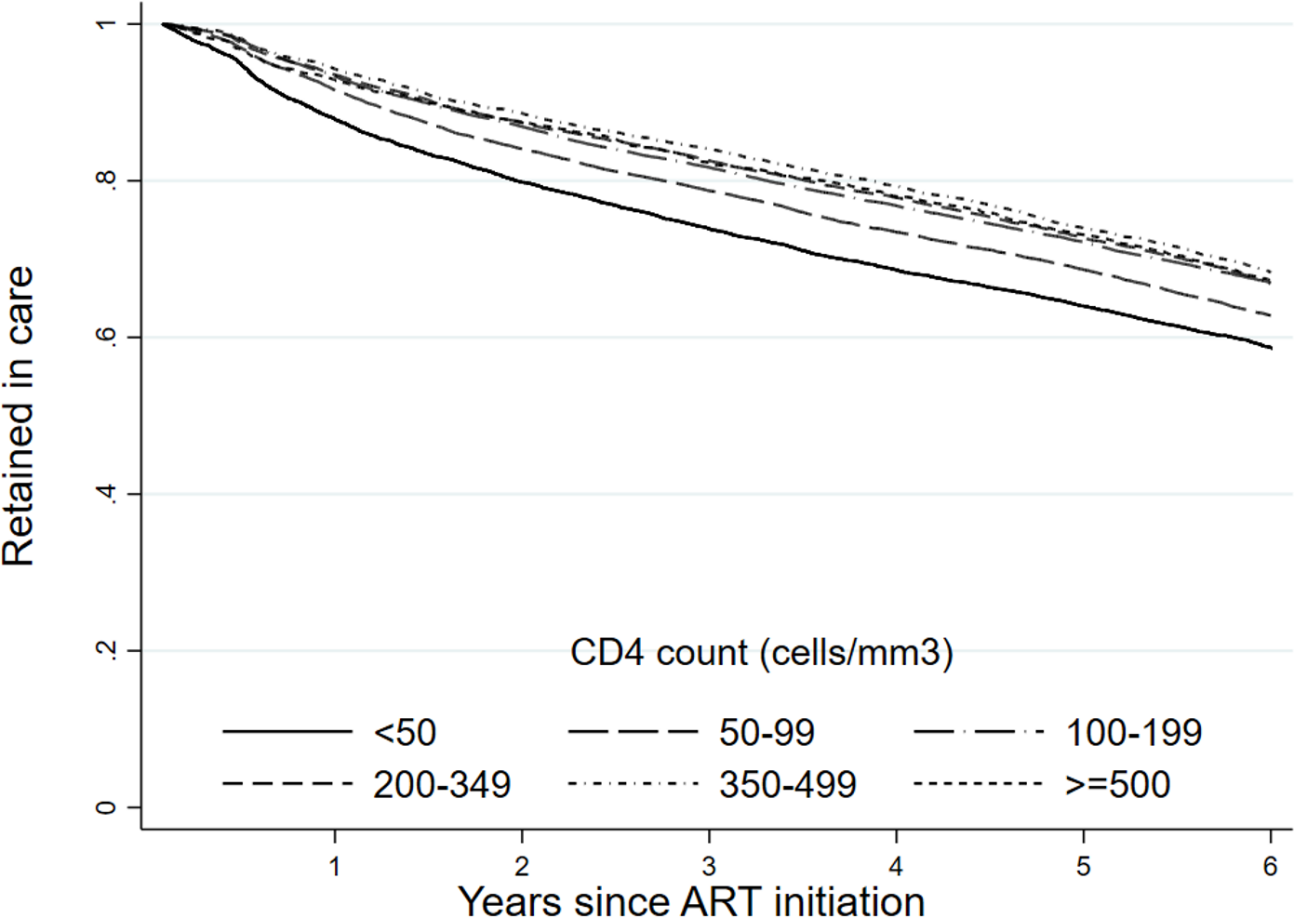
Retention by CD4 count at first viral load (retained in care on December 31, 2012) at the national level in South Africa among 55,836 patients initiating ART in 2004–2006 (retrospective definition).

**Fig 3:**
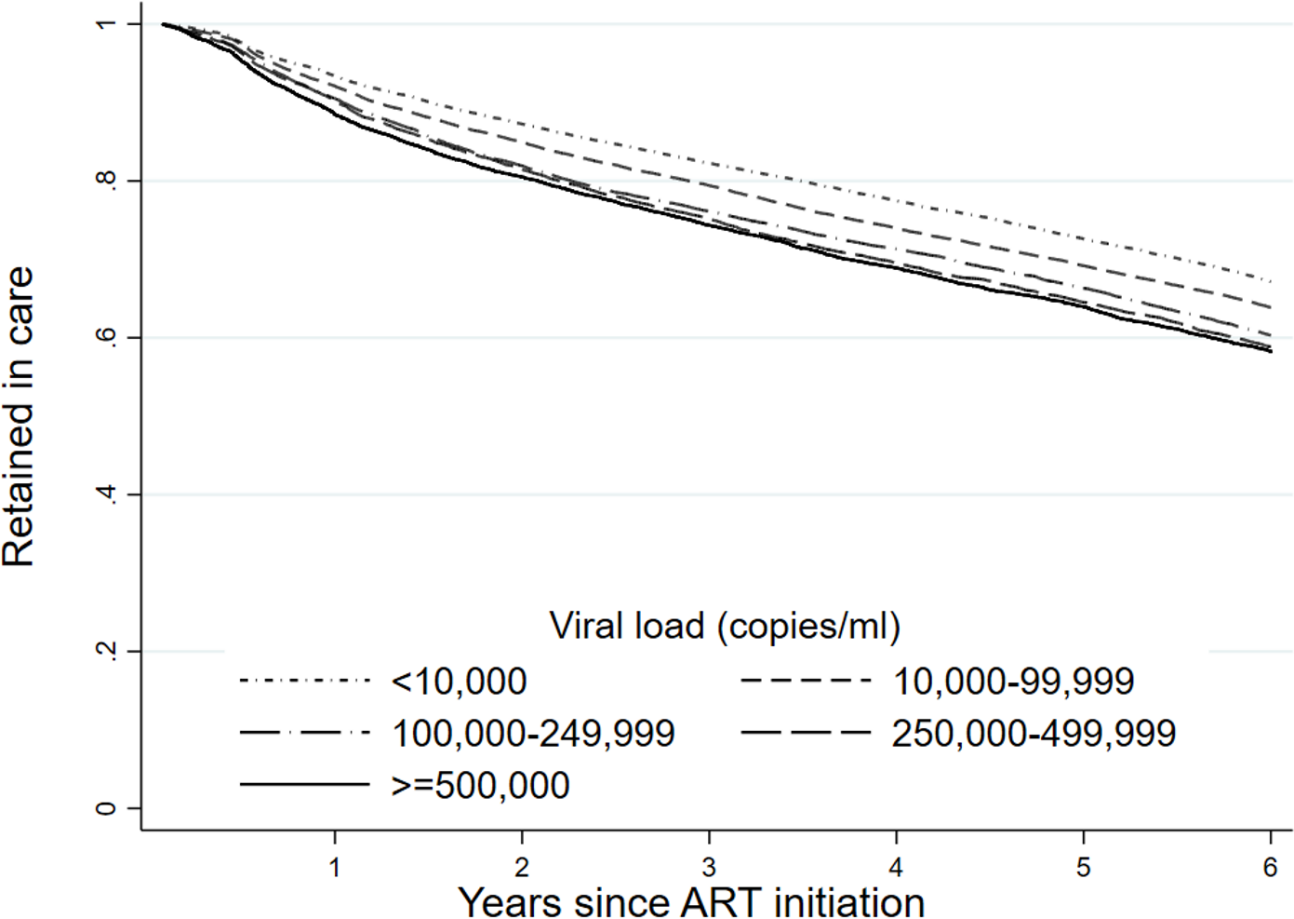
Retention by first viral load (retained in care on December 31, 2012) at the national level in South Africa among 55,836 patients initiating ART in 2004–2006 (retrospective definition).

We identified important variation in retention using the national perspective. Fig 2 shows retention stratified by CD4 count at ART initiation. Since retention includes both death and loss to follow-up, it is not surprising that retention is predicted by first CD4 count, with an 8.6 percentage point difference in national retention rate between those with a CD4 count at initiation of ≥500 cells/mm^3^ (67.3%; 95% CI: 65.2%–69.3%) and those with a CD4 count of <50 cells/mm^3^ (58.7%; 95% CI: 57.5%–59.8%) over the 6 years. We found similar associations with viral load at ART initiation (Fig 3).

**Fig 4:**
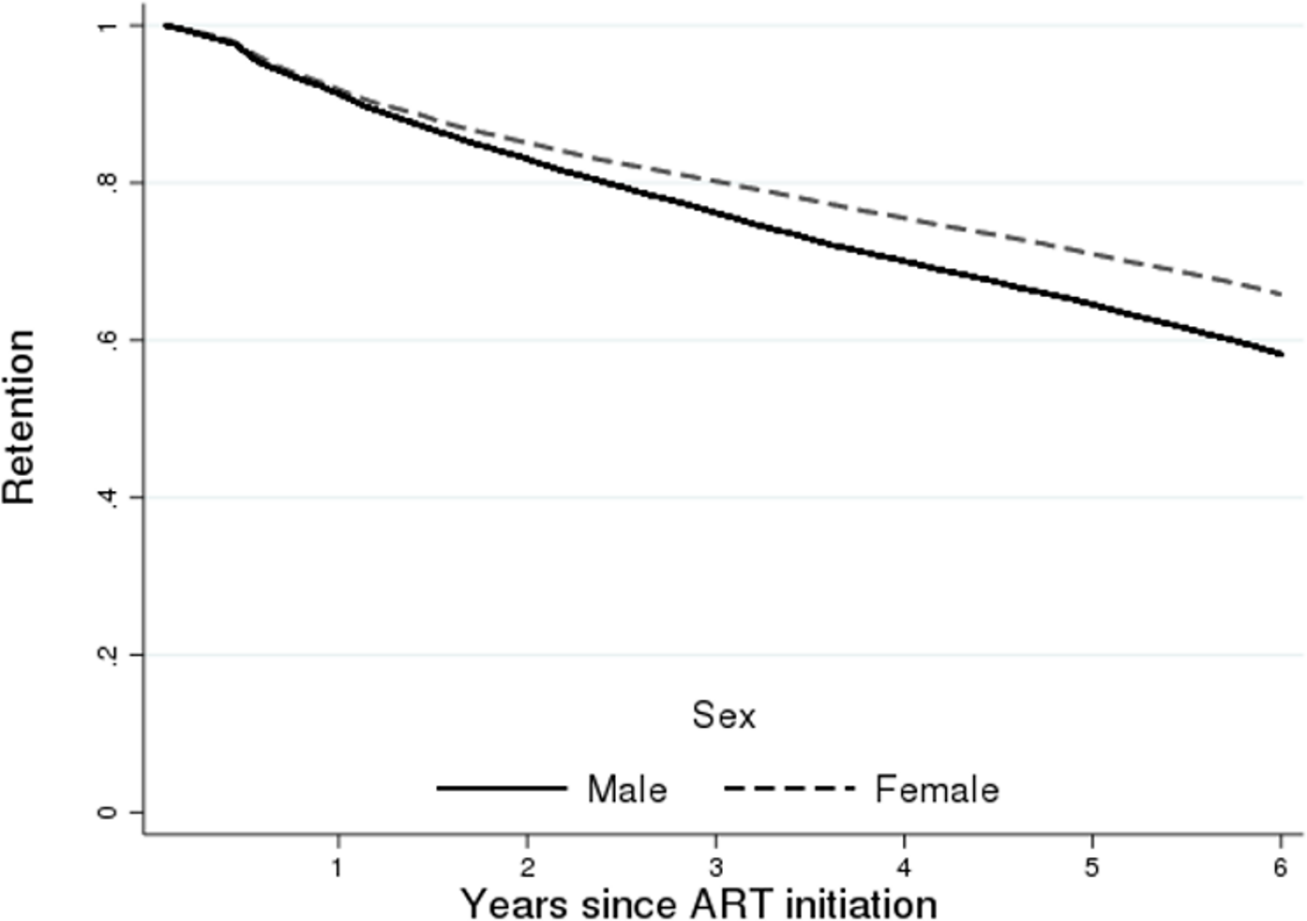
Retention (as of December 31, 2012) at the national level in South Africa among 55,836 patients initiating ART in 2004–2006 stratified by sex.

**Fig 5:**
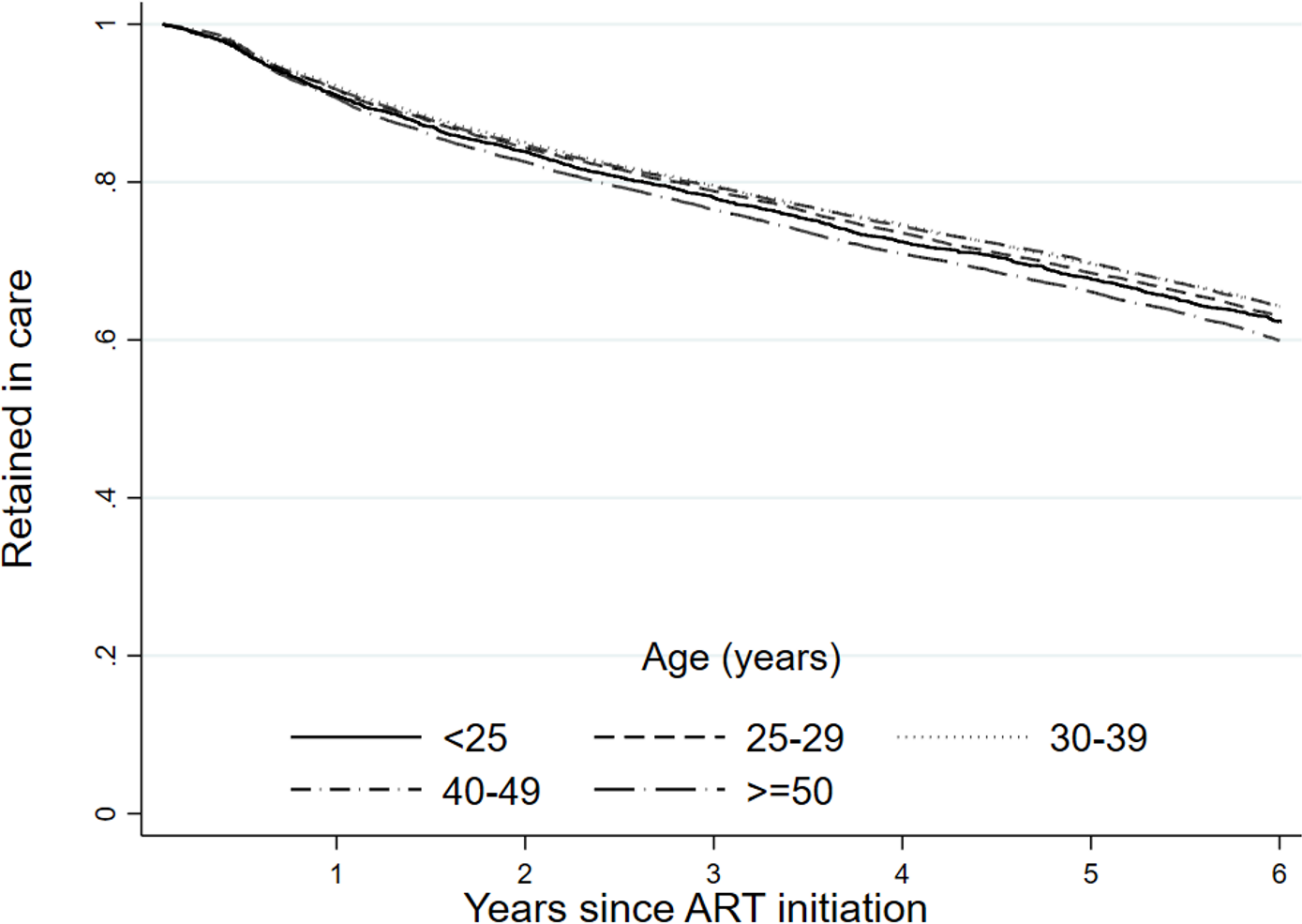
Retention (as of December 31, 2012) at the national level in South Africa among 55,836 patients initiating ART in 2004–2006 stratified by age.

**Fig 6:**
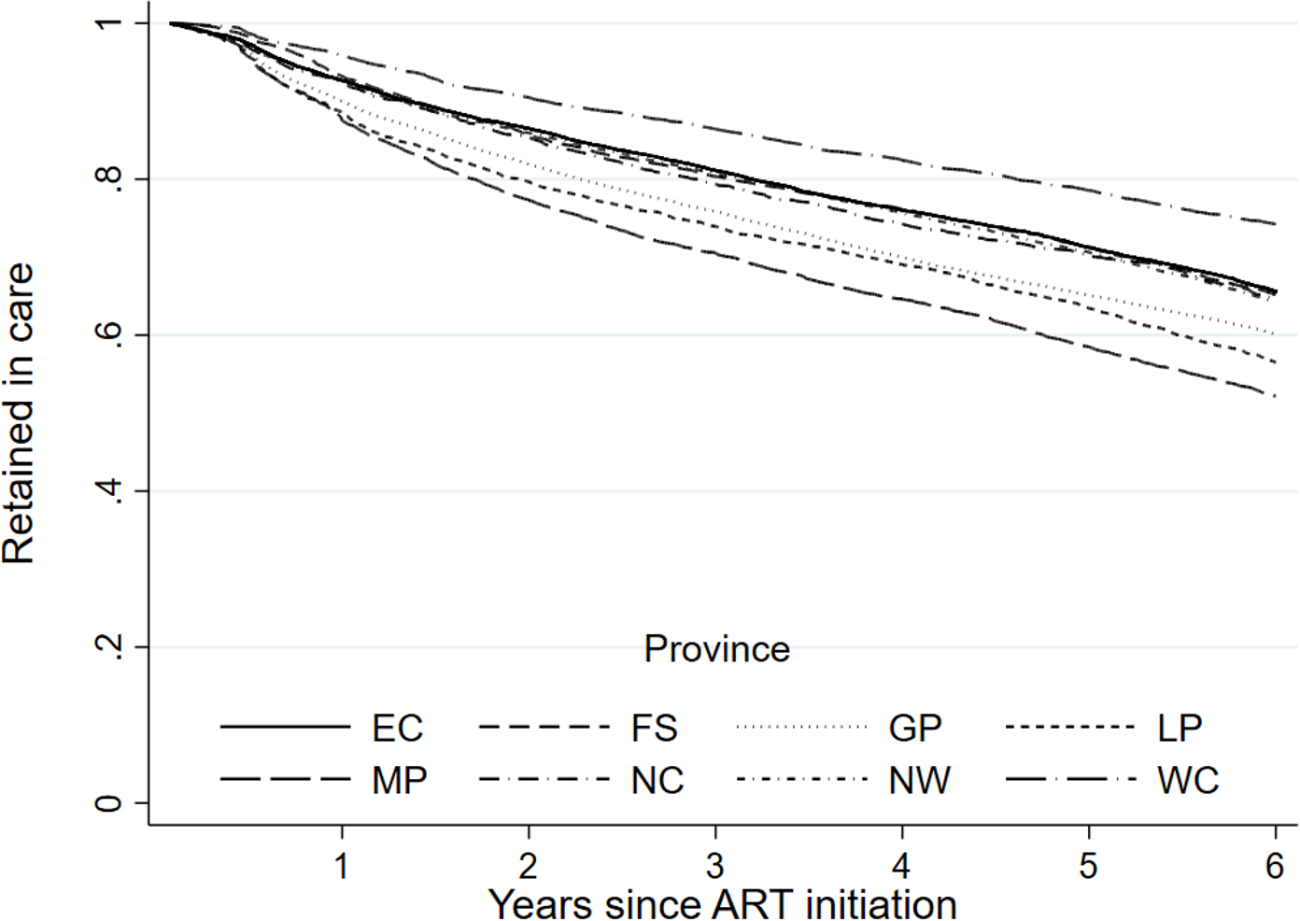
Retention (as of December 31, 2012) at the national level in South Africa among 55,836 patients initiating ART from 2004–2006 stratified by province of enrollment. EC, Eastern Cape; FS, Free State; GP, Gauteng; LP, Limpopo; MP, Mpumalanga; NC, Northern Cape; NW, North West; WC, Western Cape.

**Fig 7:**
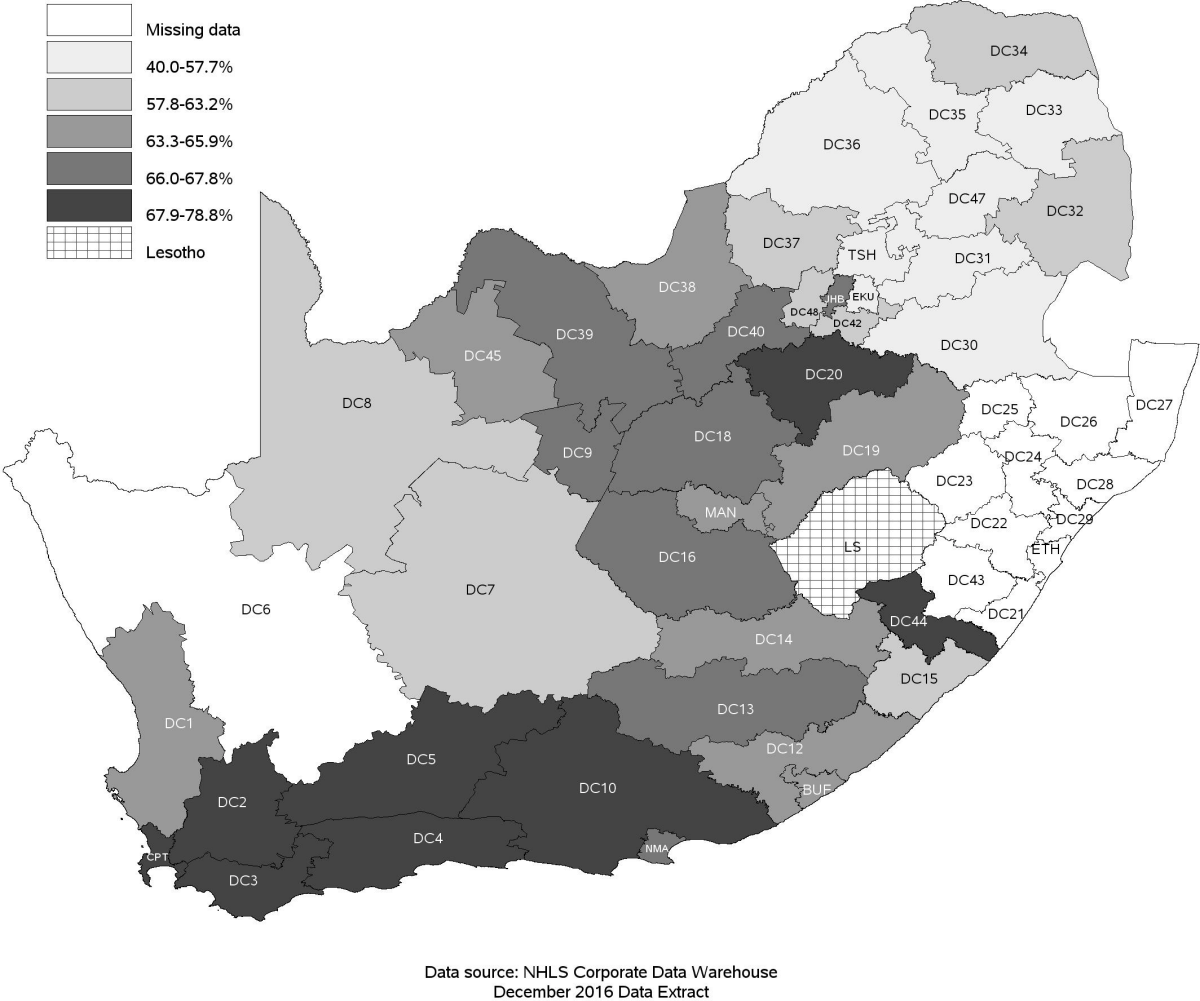
Proportion of patients retained in care on December 31, 2012, at the national level in South Africa among 55,836 patients initiating ART in 2004–2006 by district of enrollment. Proportion retained in care was categorized in quintiles. Districts: BUF, Buffalo City; CPT, City of Cape Town; DC1, West Coast; DC2, Cape Winelands; DC3, Overberg; DC4, Eden; DC5, Central Karoo; DC6, Namakwa; DC7, Pixley ka Seme; DC8, Siyanda; DC9, Frances Baard; DC10, Cacadu; DC12, Amathole; DC13, Chris Hani; DC14, Ukhahlamba; DC15, O. R. Tambo; DC16, Xhariep; DC18, Lejweleputswa; DC19, Thabo Mofutsanyana; DC20, Fezile Dabi; DC21, Ugo; DC22, UMgungundlovu; DC23, Uthukela; DC24, Umzinyathi; DC25, Amajuba; DC26, Zululand; DC27, Umkhanyakude; DC28, Uthungulu; DC29, iLembe; DC30, Gert Sibande; DC31, Nkangala; DC32, Ehlanzeni; DC33, Mopani; DC34, Vhembe; DC35, Capricorn; DC36, Waterberg; DC37, Bojanala; DC38, Ngaka Modiri Molema; DC39, Dr Ruth Segomotsi Mompati; DC40, Dr Kenneth Kaunda; DC42, Sedibeng; DC43, Sisonke; DC44, Alfred Nzo; DC45, John Taolo Gaetsewe; DC47, Greater Sekhukhune; DC48, W. Rand; EKU, Ekurhuleni; ETH, eThekwini; JHB, Johannesburg; LS, Lesotho; MAN, Mangaung; NMA, Nelson Mandela Bay Metro; TSH, City of Tshwane. NHLS, National Health Laboratory Service.

**Fig 8:**
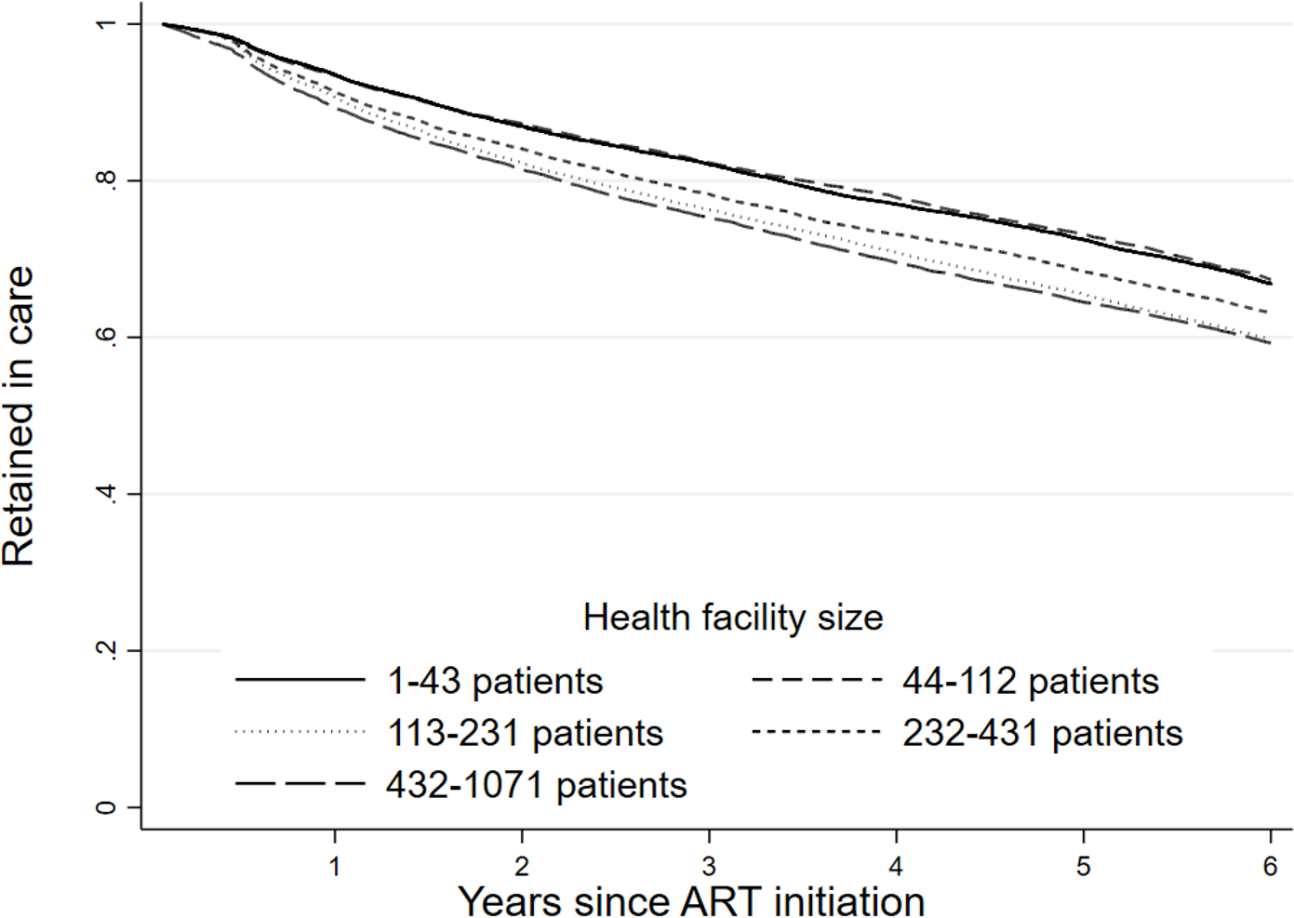
Retention (as of December 31, 2012) at the national level in South Africa among 55,836 patients initiating ART in 2004–2006 stratified by health facility size.

When looking at demographic factors, we found a small but meaningful difference in retention by sex (Fig 4). As attrition includes death, we also observed an expected association with age (Fig 5), where we saw a reduction in retention with increasing age, with those 50 years of age and older (59.9%; 95% CI: 58.9%–60.9%) having a 6.0 percentage point lower retention rate than those under 25 years (62.4%; 95% CI: 60.6%–64.1%) at 6 years of follow-up. There was also a strong difference in retention by province (Fig 6), with Western Cape (74.2%; 95% CI: 73.2%–75.2%) having a much higher retention rate, while Mpumalanga had the lowest (52.2%; 95% CI: 50.6%–53.7%), at 6 years. This is seen broken down by district in Fig 7, where retention rates are higher in many of the urban areas of the country; the Western Cape in the southwest has the highest retention, followed by Gauteng and the Eastern Cape. Retention was lower in provinces with more rural areas like Limpopo and Mpumalanga. Rates of retention at 6 years were highest at clinics with fewer patients, with a linear decline in retention from the highest quintile of clinic size to the lowest. Clinics in the lowest quintile (1 to 43 patients) had a 6-year retention of 66.9% (95% CI: 66.0%–67.7%) while clinics in the highest quintile (432 to 1,071 patients) had a 6-year retention of 59.3% (95% CI: 58.4%–60.2%) (Fig 8).

Table 2 shows how movement between clinics and between provinces occurred. Depending on the province, between 32% and 67% of all patients moved clinics at least once, with 50.3% of all patients transferring at least once overall. In all provinces, among those who had at least 1 clinic transfer, it was most common for patients to transfer to another clinic within the same province at which care was initiated. Of the 35,354 patients who were retained in care at the national level, 3,969 had a gap between viral load measures of at least 24 months. Median time between viral loads for these patients was 983 days (IQR: 826–1,304 days).

**Table 2:** Movement between clinics by province of initiation and province transferred to (*n =* 55,836).

Initial province	Never transferred, *n* (%)	Province transferred to, *n* (%)									Total
		EC	FS	GP	KZ*	LP	MP	NC	NW	WC	
EC	5,175 (46.6)	5,635 (50.6)	11 (0.10)	72 (0.64)	30 (0.27)	3 (0.03)	10 (0.09)	5 (0.04)	11 (0.10)	167 (1.50)	11,118 (19.9)
FS	1,176 (33.3)	19 (0.54)	2,269 (64.4)	30 (0.85)	2 (0.06)	2 (0.06)	5 (0.14)	9 (0.26)	10 (0.28)	3 (0.09)	3,529 (6.3)
GP	9,265 (56.9)	135 (0.83)	103 (0.63)	6,307 (38.8)	36 (0.22)	118 (0.73)	64 (0.39)	21 (0.13)	188 (1.15)	31 (0.19)	16,269 (29.1)
LP	2,257 (63.6)	0 (0.0)	2 (0.06)	61 (1.78)	3 (0.08)	1,194 (33.6)	18 (0.51)	1 (0.03)	12 (0.34)	0 (0.0)	3,546 (6.4)
MP	2,636 (68.0)	3 (0.08)	4 (0.10)	51 (1.32)	7 (0.18)	17 (0.44)	1,153 (29.7)	0 (0.0)	3 (0.08)	3 (0.08)	3,876 (6.9)
NC	625 (35.7)	14 (0.80)	11 (0.63)	22 (1.25)	0 (0.0)	3 (0.17)	0 (0.0)	1,041 (59.3)	27 (1.54)	11 (0.63)	1,754 (3.1)
NW	3,700 (42.4)	31 (0.36)	34 (0.39)	152 (1.74)	0 (0.0)	16 (0.18)	8 (0.09)	37 (0.42)	4,728 (54.3)	8 (0.09)	8,713 (15.6)
WC	2,938 (41.8)	234 (3.31)	9 (0.13)	43 (0.61)	5 (0.07)	0 (0.0)	3 (0.04)	25 (0.36)	6 (0.09)	3,770 (53.6)	7,031 (12.6)
**Total**	27,772 (49.7)	6,071 (10.9)	2,443 (4.4)	6,738 (12.1)	83 (0.15)	1,353 (2.42)	1,261 (2.26)	1,139 (2.04)	4,985 (8.92)	3,991 (7.15)	55,836

*KwaZulu-Natal did not contribute data until after 2010.

EC, Eastern Cape; FS, Free State; GP, Gauteng; KZ, KwaZulu-Natal; LP, Limpopo; MP, Mpumalanga; NC, Northern Cape; NW, North West; WC, Western Cape.

The previous analyses are not adjusted for other covariates. Adjusted hazard ratios (HRs) of national attrition are shown in Table 3. As in the Kaplan–Meier curves, we saw a dose response with CD4 count such that those with lower CD4 counts at ART initiation had higher attrition (death and loss to follow-up). Those with a first CD4 count of <50 had a 25% higher attrition (HR: 1.25; 95% CI: 1.19–1.31) than those with a CD4 count of 100–199 cells/mm^3^, while those with a viral load of ≥500,000 copies/ml had 28% more attrition than those with 1,000–9,999 copies/ml (HR: 1.28; 95% CI: 1.21–1.35). Further, we found a small but consistent dose–response increase in attrition associated with quintile of clinic size. Those in clinics in the highest quintile (432–1,071 patients) were 25% (HR 1.25; 95% CI: 1.19–1.31) more likely to experience attrition as those in the smallest clinics (1–43 patients). We also observed an association between male sex (HR 1.29; 95% CI: 1.25–1.33) and increased attrition. With respect to age, the relationship was not linear, with the oldest and youngest groups having the highest rates of attrition.

**Table 3:** Adjusted* hazard ratios of predictors of national-level attrition in South Africa’s national ART program (*n =* 55,836).

Variable	Number of events (% attrition)	Person-years	Crude HR (95% CI)	Adjusted HR (95% CI)
**CD4 count (cells/mm**^**3**^**) at ART initiation**				
≥500	659 (32.7)	32,957.2	0.98 (0.90–1.06)	1.01 (0.93–1.10)
350–499	1,106 (31.7)	38,044.8	0.94 (0.88–1.00)	0.99 (0.93–1.06)
200–350	3,102 (31.7)	88,911.0	0.99 (0.95–1.03)	1.04 (0.99–1.08)
100–199	5,964 (33.2)	46,795.6	Reference	Reference
50–99	2,961 (37.2)	17,605.0	1.16 (1.11–1.21)	1.10 (1.06–1.16)
<50	3,001 (41.3)	10,034.5	1.36 (1.30–1.42)	1.25 (1.19–1.31)
**Viral load (copies/ml) at ART initiation**				
1,000–9,999	6,318 (32.8)	95,701.5	Reference	Reference
10,000–99,999	5,900 (36.2)	78,628.8	1.14 (1.10–1.18)	1.07 (1.03–1.12)
100,000–249,999	3,230 (39.7)	37,962.6	1.29 (1.23–1.34)	1.20 (1.14–1.26)
250,000–499,999	2,203 (41.1)	24,667.1	1.35 (1.29–1.42)	1.25 (1.18–1.32)
≥500,000	2,832 (41.8)	30,855.4	1.39 (1.33–1.45)	1.28 (1.21–1.35)
**Age (years) at ART initiation**				
<25	1,080 (37.6)	13,632.6	0.92 (0.86–0.99)	1.10 (1.02–1.19)
25–29.9	2,856 (37.1)	36,850.5	0.90 (0.86–0.95)	1.02 (0.97–1.08)
30–39.9	8,167 (35.7)	110,568.0	0.86 (0.83–0.90)	0.91 (0.87–0.96)
40–49.9	4,934 (35.8)	66,612.1	0.86 (0.83–0.90)	0.90 (0.85–0.94)
≥50	3,446 (40.1)	40,152.3	Reference	Reference
**Sex**				
Female	12,557 (34.2)	85,192.5	Reference	Reference
Male	7,668 (41.8)	178,890.5	1.28 (1.24–1.32)	1.29 (1.25–1.33)
**Province**				
Gauteng	3,828 (34.4)	54,571.1	Reference	Reference
Eastern Cape	1,226 (34.7)	17,305.0	0.82 (0.78–0.85)	0.93 (0.90–0.97)
Free State	6,487 (39.9)	75,368.2	0.82 (0.77–0.88)	0.88 (0.82–0.95)
Limpopo	1,543 (43.5)	16,074.5	1.12 (1.06–1.18)	1.28 (1.20–1.36)
Mpumalanga	1,854 (47.8)	16,870.0	1.28 (1.21–1.34)	1.32 (1.24–1.41)
Northern Cape	620 (35.4)	8,495.0	0.85 (0.78–0.92)	0.97 (0.89–1.06)
North West	3,112 (35.7)	42,509.2	0.85 (0.82–0.89)	0.95 (0.90–0.99)
Western Cape	1,813 (25.8)	36,622.4	0.58 (0.55–0.61)	0.66 (0.62–0.70)
**Clinic size quintile**				
1–43 patients	3,775 (33.2)	56,458.0	Reference	Reference
44–112 patients	3,690 (32.8)	55,938.0	0.99 (0.94–1.03)	0.98 (0.93–1.03)
113–231 patients	4,360 (40.1)	50,815.0	1.28 (1.23–1.34)	1.14 (1.08–1.20)
232–431 patients	4,121 (36.9)	53,301.6	1.16 (1.11–1.21)	1.16 (1.11–1.22)
432–1,071 patients	4,537 (40.7)	51,302.8	1.32 (1.27–1.38)	1.25 (1.19–1.31)

*Adjusted for all other factors in the model.

CI, confidence interval; HR, hazard ratio.

## Discussion

In this national assessment of retention in South Africa’s HIV program, we found that among patients starting ART in 2004–2006, national retention was 63.3% (95% CI: 62.9%–63.7%) at 6 years, more than twice the proportion retained at the initiating clinic (29.1%; 95% CI: 28.7%–29.5%). This 34.2 percentage point difference suggests that considering retention only from the perspective of the initiating clinic, the approach most commonly used to estimate retention in clinical cohort studies, strongly overstates attrition.

We note that our estimates—which are based on laboratory monitoring data—may understate retention if there are gaps in clinical adherence to national monitoring protocols. Nevertheless, the relative difference in retention observed when moving from a facility to a national perspective suggests that existing facility-based estimates of patient retention may be too low. Further, even in our sensitivity analysis in which we used a prospective attrition definition (first time to attrition), the differences were still meaningful, with patients more than twice as likely to be retained within the health system as at the initiating clinic at 6 years. The lower estimate of national retention obtained using a prospective definition most likely reflects the fact that gaps in care are common, though this is also likely influenced by imperfections in linking laboratory results.

While there are no systematic reviews with sufficient follow-up to compare our findings to, we can compare them to our 2015 retention review through 5 years [42], but only from a clinic perspective. In the review, 5-year retention was 60%, versus 39% here, using a clinic perspective, about a 20 percentage point difference. It is not clear why this difference occurred, but we can say that our current results reflect a countrywide view of the clinic perspective, while the review likely reflected a highly selected set of clinics with the ability to report on and publish retention findings. An analysis of data using TIER.net (South Africa’s electronic patient record system) found 8-year retention at about 60% [23] for those initiated on ART in 2004–2005, whereas our cohort reached 63% at 6 years. The TIER.net analysis has limited ability to account for silent transfers and patients who leave care and then return, while our study does not suffer this limitation. While our estimates are for the early years of the national program, we are currently developing an approach to identify ART initiation [31] in later cohorts, when ART initiation viral load testing was not done. This will allow us to repeat this analysis in later cohorts to determine if these patterns hold over time.

As our results reflect retention for all patients, even those who transferred clinics, we were also able to assess retention from the clinic perspective (which often would not have been informed about transfers). Our 6-year retention results accounting for patient movement differ dramatically from our results assuming that a patient who left a clinic was lost, with about a 30 percentage point difference between them. We note that this analysis does not account for known formal transfers, which clinics typically would not count as attrition but we did. Still, we found that movement between clinics was common. This is important for reporting on overall retention as well as numbers initiating ART, as silent transfers are often treated by clinics as new patients, biasing retention estimates [23].

It is not clear at this point whether our results generalize to other countries, and there is reason to believe they may not tell us what is occurring outside South Africa. South Africa’s national program includes some very large clinics in urban areas, which may drive the results to a certain extent, and clinic practices in South Africa likely differ from those in other countries. In addition, we do not yet know if these findings represent what is occurring in South Africa’s ART program currently, as scale-up of treatment may mean that transfers are more common now (as patients have more options) but may also mean less attrition from a national perspective as care may be more convenient to patients. In addition, shifts to decentralizing HIV care and moving care out of stand-alone clinics and into primary care may also impact generalizability. As we refine our methodology to allow us to identify ART initiation after viral load testing at ART initiation was discontinued, we will be able to assess changes in retention over time.

Our findings that CD4 count and viral load at entry into care are predictive of attrition are not surprising. Many prior cohorts have found similar patterns [43,44], and the findings are logical given that attrition includes mortality, which is known to increase with declining immune functioning. Patients with CD4 counts under 50 cells/mm^3^ had the highest attrition while those in each of the CD4 categories ≥100 cells/mm^3^ had roughly similar attrition to each other. The impact of mortality may be muted by the impact of higher loss to follow-up among those who are younger. We also found a relationship between clinic size and attrition, with higher attrition seen at larger clinics. We do not know for sure the reason for this, but we do suspect that this represents a combination of factors including a rural–urban divide, with larger clinics in urban areas, while also representing the impact of clinic crowding and the fact that large clinics likely see complicated cases that are at higher risk of attrition and early mortality. We also found some differences by age, with highest attrition among the oldest and youngest patients. This likely reflects mortality among the oldest patients and loss to follow-up among the younger patients, but without specific mortality data we cannot say for sure. Differences were small by sex, with somewhat higher attrition among men, but to the extent that they reflect real patterns, they probably reflect differences in care seeking behaviors between men and women.

Our results highlight the impact of silent transfers on retention estimates. One approach proposed for dealing with silent transfers is tracing methods that use samples of patients lost and that apply the resulting proportion of patients in care at a new clinic from the sample to the full cohort [16,26,45]. Another approach, which could work in South Africa, has been to use the national population register, which records deaths [28,43], to identify which patients lost to follow-up have died or to use approximations to adjust for death among those lost [27]. The former, which allows categorizing patients into those lost, in care, and who died, relies strongly on those being found being a random sample of all patients lost. The latter only allows interrogating the impact of those who died, but cannot separate those lost from the initiating site who are still in care at another clinic and those not in care anywhere. While these methods all improve cohort analyses, they can only give summary proportions of retention and cannot describe retention over time since initiation. As such, our approach may provide better estimates than these previously proposed approaches. Still, approaches to ensuring smooth transition of care between sites are critical for improving treatment outcomes. Use of a national patient identifier could help improve data integration and support successful transitions across treatment sites. In addition, as our approach is less prone to the overcounting of patients that occurs with other approaches to reporting numbers on treatment, it may also be more accurate in counting patients who are lost to care. Until a unique patient identifier is put into place that allows for tracking of patients across clinical treatment sites in South Africa (and other programs), such approaches will be able to provide a critical additional approach to measuring retention across clinical sites.

Another key finding in our study is that movement between clinics over time was quite common in this population. We found that nearly half of all patients enrolled in the early years of the program had at least 1 transfer. Most of those transfers were within the same province. This high rate of transfers makes sense for several reasons. First, as the national HIV care and treatment program developed, more clinics opened, particularly in urban areas, giving patients more choices for where to seek care. Patients might move to a different clinic farther from their community to allow some level of anonymity, or to a clinic closer for more convenience. Second, migration is common in South Africa for work-related reasons, particularly among men, and it is common among women at the time of pregnancy, when women may return to a family home either during or after giving birth in order to receive support for caring for the child. In terms of movement between provinces, much of this movement reflects movement to areas with higher population density and opportunities for work, especially Gauteng province, where Johannesburg and Pretoria reside. It will be important for future research to attempt to determine the reason for this movement to ensure a high rate of linkage to care as movement occurs.

We note that our results only apply to patients who received viral load testing at baseline as recommended by treatment guidelines at the time. Other patients who initiated ART but did not have a viral load test would not be observed. During the time, government data suggest that 239,000 patients were receiving ART in early 2006 [46] compared to the roughly 55,000 that we identified, and 118,000 in our sensitivity analyses. Even accounting for our exclusion of KwaZulu-Natal, the province with the largest number of HIV-infected patients, and for the fact that the government estimates could include some double counting of patients who left one clinic and reenrolled at another, our results clearly do not include all patients. It is not clear who would have received viral load testing at ART initiation or if this would impact retention in HIV care.

Our data have some important limitations. First, our probabilistic matching to create our cohort was subject to both over- and undermatching, which may lead to over- or underestimation of retention. However, our primary results excluded patients linked to laboratory results with low confidence, and our results were robust to the inclusion of these patients. Second, we used information on clinic to determine the match probability, meaning that, all things being equal, laboratory tests from the same facility were more likely to be linked to each other, which biases estimates of movement between clinics downward. Third, we focused only on the 2004–2006 cohort in order to describe retention since ART initiation. Trends may change with later cohorts as more clinics were in place, stigma was reduced, and treatment initiation protocols were different. It is also not clear that such patterns will continue to hold as the program matured and CD4 count thresholds increased and, more recently, were removed. Healthier patients enrolling into programs and initiating ART may have different retention patterns than those initiating with very low CD4 counts. In addition, it is not clear that the mobility patterns we observed will have continued as the program and the countrywide migration patterns changed. Future analyses could shed light on this if a validated approach can be identified to allow identification of ART initiation in this laboratory cohort after viral load testing at staging was no longer recommended in South Africa. Fourth, because we defined retention based on laboratory values and not visits, it is possible patients could have been in care but not receiving laboratory monitoring. These patients would be considered lost when they should be considered retained in care. This likely accounts for differences between our cohort and other cohorts and TIER.net. Fifth, we were not able to include data from KwaZulu-Natal, a province with large numbers of patients on treatment. Because HIV prevalence is so high in this province, our numbers are lower than would be expected in a national analysis. In addition, because migration is common in this province, our estimate of movement between clinics may be underestimated. Sixth, we cannot distinguish between loss to follow-up and death. Seventh, we cannot guarantee that all clinics in South Africa were using viral load testing at ART staging as guidelines stipulated. If some clinics were ignoring the viral load at staging, we might be identifying the first viral load on treatment rather than the date of ART initiation; if so, we might be missing some attrition that occurred between ART initiation and first viral load on therapy. Further, if a patient was particularly ill, it is possible that a clinician may have initiated the patient onto treatment without taking the ART initiation viral load. Our results also featured a higher than expected number of patients with CD4 counts at or above 200 cells/mm^3^ (the eligibility cutoff at the time) at the time of ART initiation. Some of these patients may have had a first viral load observed in the database when they were already on ART, either because no baseline viral load was taken at initiation or because of possible undermatching in the probabilistic linkage, in spite of high matching sensitivity. Because our analysis was approved only on de-identified data, we could not validate first viral load as a proxy for ART initiation. Taken together, however, these limitations suggest that our analyses did not start at ART initiation for all patients, but it is unclear what direction this would bias results. We also note that our definitions of cycling in and out of care and transfer between sites are limited in that we cannot observe clinic visits and as such we can only observe large gaps in care. This analysis is also limited by the fact that we cannot distinguish between movement between sites and patients who seek care at more than 1 site (e.g., 1 site for HIV care and another for primary healthcare, where they may have a hemoglobin test that appears as movement between clinics). Finally, our primary analysis includes patients with 2 viral loads to ensure initiation, but this could overstate retention, excluding in particular those patients who were lost to follow-up shortly after initiating therapy. Our sensitivity analyses show that although the overall levels of retention are sensitive to the inclusion/exclusion of patients with just 1 viral load, the substantive conclusions are largely the same.

### Conclusion

The world over, retention in HIV care is suboptimal, but few analyses have shown this on a national scale. Our results show that, compared to clinic-only perspectives, national retention is likely substantially higher. This is because patients exiting care from the initiating clinic may reenter care elsewhere (i.e., silent transfers) and appear as lost to follow-up when they are still in care. These results are encouraging as they suggest patients who left care after starting treatment early in the epidemic often returned to care at later points in time. Further research will be needed to understand the care trajectories and long-run health outcomes of patients who cycle into and out of care at multiple facilities.

## Supporting information

S1 STROBE Checklist STROBE checklist. (DOC)

S1 Appendix Effect of patient transfer on retention estimates overall in South Africa from ART initiation with attrition defined prospectively as first time without a lab test for 24 months. (DOCX)

S2 Appendix Effect of patient transfer on retention estimates overall in South Africa from ART initiation in the lower quality matched cohort (*n* = 72,256). (DOCX)

S3 Appendix Effect of patient transfer on retention estimates overall in South Africa from ART initiation amongst those with 2 viral loads plus a hemoglobin or ALT measurement within 90 days before or after first viral load (*n* = 19,415). (DOCX)

S4 Appendix Effect of patient transfer on retention estimates overall in South Africa from ART initiation including all patients with at least 1 viral load (*n* = 118,720). (DOCX)

S5 Appendix Effect of patient transfer on retention estimates overall in South Africa from ART initiation among 33,201 patients initiating ART in 2004–2006 with a CD4 count < 200 cells/mm3 and retention defined as retained in care on December 31, 2012. (DOCX)

## Abbreviations

ALT: alanine transaminase
ART: antiretroviral therapy
HR: hazard ratio
NHLS: National Health Laboratory Service
WHO: World Health Organization
