# Correction: Estimating retention in HIV care accounting for patient transfers: A national laboratory cohort study in South Africa

**DOI:** 10.1371/journal.pmed.1002643

**Published:** 2018-08-10

**Authors:** Matthew P. Fox, Jacob Bor, Alana T. Brennan, William B. MacLeod, Mhairi Maskew, Wendy S. Stevens, Sergio Carmona

A grant supporting the work in this article was mistakenly omitted from the original funding statement. The funding statement in full should read as below:

“This work was supported by grants 1R01AI115979-01, 1K01MH105320-01A1 and 5T32AI052074-13 from the National Institutes of Health. WBM and SC were supported by USAID through Cooperative Agreement AID- 674-A-12-0020 from the United States Agency for International Development (USAID). The funders had no role in study design, data collection and analysis, decision to publish, or preparation of the manuscript.”
