# Supplementary material for: Estimating retention in HIV care accounting for patient transfers: A national laboratory cohort study in South Africa
Source: PLoS Med. 2018 Jun 11;15(6):e1002589. doi: 10.1371/journal.pmed.1002589 (PMC5995345; doi:10.1371/journal.pmed.1002589)
Supplement: S1 STROBE Checklist — (DOC) [file pmed.1002589.s001.doc]

**S1 STROBE Checklist**

|  | Item No | Recommendation |
| --- | --- | --- |
| **Title and abstract** | 1 | (*a*) Indicate the study’s design with a commonly used term in the title or the abstract **title** |
|  |  | (*b*) Provide in the abstract an informative and balanced summary of what was done and what was found - **abstract** |
| Introduction | | |
| Background/rationale | 2 | Explain the scientific background and rationale for the investigation being reported – **introduction all paragraphs** |
| Objectives | 3 | State specific objectives, including any prespecified hypotheses **introduction last paragraph** |
| Methods | | |
| Study design | 4 | Present key elements of study design early in the paper **methods** |
| Setting | 5 | Describe the setting, locations, and relevant dates, including periods of recruitment, exposure, follow-up, and data collection **methods paragraphs 1-7** |
| Participants | 6 | (*a*) Give the eligibility criteria, and the sources and methods of selection of participants. Describe methods of follow-up **methods paragraphs 4-5** |
|  |  | (*b*) For matched studies, give matching criteria and number of exposed and unexposed **N/A** |
| Variables | 7 | Clearly define all outcomes, exposures, predictors, potential confounders, and effect modifiers. Give diagnostic criteria, if applicable **methods paragraphs 5-8** |
| Data sources/ measurement | 8* | For each variable of interest, give sources of data and details of methods of assessment (measurement). Describe comparability of assessment methods if there is more than one group **methods paragraph 6-8** |
| Bias | 9 | Describe any efforts to address potential sources of bias **methods paragraph 7-8** |
| Study size | 10 | Explain how the study size was arrived at **methods paragraph 9** |
| Quantitative variables | 11 | Explain how quantitative variables were handled in the analyses. If applicable, describe which groupings were chosen and why **methods paragraph 9** |
| Statistical methods | 12 | (*a*) Describe all statistical methods, including those used to control for confounding **methods paragraph 9** |
|  |  | (*b*) Describe any methods used to examine subgroups and interactions **methods paragraph 9** |
|  |  | (*c*) Explain how missing data were addressed **methods paragraph 9** |
|  |  | (*d*) If applicable, explain how loss to follow-up was addressed **N/A as this was our outcome** |
|  |  | (*e*) Describe any sensitivity analyses **methods paragraph 6-8** |
| Results | | |
| Participants | 13* | (a) Report numbers of individuals at each stage of study—eg numbers potentially eligible, examined for eligibility, confirmed eligible, included in the study, completing follow-up, and analysed **results paragraph 1** |
|  |  | (b) Give reasons for non-participation at each stage **N/A as this was the outcome** |
|  |  | (c) Consider use of a flow diagram **N/A as we did not have losses given the outcome** |
| Descriptive data | 14* | (a) Give characteristics of study participants (eg demographic, clinical, social) and information on exposures and potential confounders **Table 1** |
|  |  | (b) Indicate number of participants with missing data for each variable of interest **Table 1** |
|  |  | (c) Summarise follow-up time (eg, average and total amount) **Results paragraph 1** |
| Outcome data | 15* | Report numbers of outcome events or summary measures over time **Results paragraph 1-2 and table 1** |
| Main results | 16 | (*a*) Give unadjusted estimates and, if applicable, confounder-adjusted estimates and their precision (eg, 95% confidence interval). Make clear which confounders were adjusted for and why they were included **Tables and Results paragraph 1-7** |
|  |  | (*b*) Report category boundaries when continuous variables were categorized **provided in tables** |
|  |  | (*c*) If relevant, consider translating estimates of relative risk into absolute risk for a meaningful time period **N/A for primary outcomes which are risks** |
| Other analyses | 17 | Report other analyses done—eg analyses of subgroups and interactions, and sensitivity analyses **Results paragraph 7** |
| Discussion | | |
| Key results | 18 | Summarise key results with reference to study objectives **Discussion paragraph 1 and 2** |
| Limitations | 19 | Discuss limitations of the study, taking into account sources of potential bias or imprecision. Discuss both direction and magnitude of any potential bias **Discussion paragraph 10** |
| Interpretation | 20 | Give a cautious overall interpretation of results considering objectives, limitations, multiplicity of analyses, results from similar studies, and other relevant evidence **Discussion paragraphs 1-5** |
| Generalisability | 21 | Discuss the generalisability (external validity) of the study results **Discussion paragraph 5** |
| Other information | | |
| Funding | 22 | Give the source of funding and the role of the funders for the present study and, if applicable, for the original study on which the present article is based **Provided to the journal** |

*Give information separately for exposed and unexposed groups.

**Note:** An Explanation and Elaboration article discusses each checklist item and gives methodological background and published examples of transparent reporting. The STROBE checklist is best used in conjunction with this article (freely available on the Web sites of PLoS Medicine at http://www.plosmedicine.org/, Annals of Internal Medicine at http://www.annals.org/, and Epidemiology at http://www.epidem.com/). Information on the STROBE Initiative is available at http://www.strobe-statement.org.
