# Supplementary material for: Estimating retention in HIV care accounting for patient transfers: A national laboratory cohort study in South Africa
Source: PLoS Med. 2018 Jun 11;15(6):e1002589. doi: 10.1371/journal.pmed.1002589 (PMC5995345; doi:10.1371/journal.pmed.1002589)
Supplement: S1 Appendix — (DOCX) [file pmed.1002589.s002.docx]

**S1 Appendix. Effect of patient transfer on retention estimates overall in South Africa from ART initiation with attrition defined prospectively as first time without a lab test for 24 months.**

|  | *Interval*  *(years)* | *Beginning*  *N* | *N*  *Attrition* | *Retained* | *95% Confidence*  *Interval* |
| --- | --- | --- | --- | --- | --- |
| **National retention** | 0-1 | 55836 | 5780 | 0.90 | 0.89-0.91 |
|  | 1-2 | 50056 | 5531 | 0.80 | 0.79-0.80 |
|  | 2-3 | 44525 | 4291 | 0.72 | 0.71-0.72 |
|  | 3-4 | 40234 | 3983 | 0.65 | 0.64-0.65 |
|  | 4-5 | 36251 | 3604 | 0.58 | 0.58-0.59 |
|  | 5-6 | 32647 | 3255 | 0.53 | 0.52-0.53 |
| **Clinic**  **retention** | 0-1 | 55836 | 7974 | 0.86 | 0.85-0.86 |
|  | 1-2 | 47862 | 7732 | 0.72 | 0.71-0.72 |
|  | 2-3 | 40130 | 6190 | 0.61 | 0.60-0.61 |
|  | 3-4 | 33940 | 6270 | 0.50 | 0.49-0.50 |
|  | 4-5 | 27670 | 6161 | 0.39 | 0.38-0.39 |
|  | 5-6 | 21509 | 5257 | 0.29 | 0.28-0.29 |
