# Supplementary material for: Estimating retention in HIV care accounting for patient transfers: A national laboratory cohort study in South Africa
Source: PLoS Med. 2018 Jun 11;15(6):e1002589. doi: 10.1371/journal.pmed.1002589 (PMC5995345; doi:10.1371/journal.pmed.1002589)
Supplement: S2 Appendix — (DOCX) [file pmed.1002589.s003.docx]

**S2 Appendix. Effect of patient transfer on retention estimates overall in South Africa from ART initiation with in the lower quality matched cohort. (N=72,256)**

|  | *Interval*  *(years)* | *Beginning*  *N* | *N*  *Attrition* | *Retained* | *95% Confidence*  *Interval* |
| --- | --- | --- | --- | --- | --- |
| **National retention** | 0-1 | 72256 | 5327 | 0.93 | 0.92-0.93 |
|  | 1-2 | 66929 | 4718 | 0.86 | 0.86-0.86 |
|  | 2-3 | 62211 | 3767 | 0.81 | 0.81-0.81 |
|  | 3-4 | 58444 | 3542 | 0.76 | 0.76-0.76 |
|  | 4-5 | 54902 | 3488 | 0.71 | 0.71-0.71 |
|  | 5-6 | 51414 | 4017 | 0.66 | 0.65-0.66 |
| **Clinic**  **retention** | 0-1 | 72256 | 9719 | 0.87 | 0.86-0.87 |
|  | 1-2 | 62537 | 9866 | 0.73 | 0.73-0.73 |
|  | 2-3 | 52671 | 8089 | 0.62 | 0.61-0.62 |
|  | 3-4 | 44582 | 8338 | 0.50 | 0.50-0.51 |
|  | 4-5 | 36244 | 8203 | 0.39 | 0.38-0.39 |
|  | 5-6 | 28041 | 6951 | 0.29 | 0.29-0.30 |
