# Supplementary material for: Estimating retention in HIV care accounting for patient transfers: A national laboratory cohort study in South Africa
Source: PLoS Med. 2018 Jun 11;15(6):e1002589. doi: 10.1371/journal.pmed.1002589 (PMC5995345; doi:10.1371/journal.pmed.1002589)
Supplement: S3 Appendix — (DOCX) [file pmed.1002589.s004.docx]

**S3 Appendix. Effect of patient transfer on retention estimates overall in South Africa from ART initiation amongst those with two viral loads plus a hemoglobin or ALT measurement within 90 days before or after first viral load (N=19,415)**

|  | *Interval*  *(years)* | *Beginning*  *N* | *N*  *Attrition* | *Retained* | *95% Confidence*  *Interval* |
| --- | --- | --- | --- | --- | --- |
| **National retention** | 0-1 | 19415 | 1841 | 0.91 | 0.90-0.91 |
|  | 1-2 | 17574 | 1521 | 0.83 | 0.82-0.83 |
|  | 2-3 | 16053 | 1081 | 0.77 | 0.77-0.78 |
|  | 3-4 | 14972 | 990 | 0.72 | 0.71-0.73 |
|  | 4-5 | 13982 | 1005 | 0.67 | 0.66-0.68 |
|  | 5-6 | 12977 | 1026 | 0.62 | 0.61-0.62 |
| **Clinic**  **retention** | 0-1 | 19415 | 3018 | 0.84 | 0.84-0.85 |
|  | 1-2 | 16397 | 2903 | 0.70 | 0.69-0.70 |
|  | 2-3 | 13494 | 2239 | 0.58 | 0.57-0.59 |
|  | 3-4 | 11255 | 2276 | 0.46 | 0.46-0.47 |
|  | 4-5 | 8979 | 2213 | 0.35 | 0.34-0.36 |
|  | 5-6 | 6766 | 1819 | 0.25 | 0.25-0.26 |
