# Supplementary material for: Estimating retention in HIV care accounting for patient transfers: A national laboratory cohort study in South Africa
Source: PLoS Med. 2018 Jun 11;15(6):e1002589. doi: 10.1371/journal.pmed.1002589 (PMC5995345; doi:10.1371/journal.pmed.1002589)
Supplement: S4 Appendix — (DOCX) [file pmed.1002589.s005.docx]

**S4 Appendix. Effect of patient transfer on retention estimates overall in South Africa from ART including all patients with at least 1 viral load (N=118,720)**

|  | *Interval*  *(years)* | *Beginning*  *N* | *N*  *Attrition* | *Retained* | *95% Confidence*  *Interval* |
| --- | --- | --- | --- | --- | --- |
| **National retention** | 0-1 | 118720 | 47677 | 0.60 | 0.60-0.60 |
|  | 1-2 | 71043 | 6708 | 0.54 | 0.54-0.54 |
|  | 2-3 | 64335 | 4870 | 0.50 | 0.50-0.50 |
|  | 3-4 | 59465 | 4407 | 0.46 | 0.46-0.47 |
|  | 4-5 | 55058 | 4061 | 0.43 | 0.43-0.43 |
|  | 5-6 | 50997 | 4495 | 0.39 | 0.39-0.39 |
| **Clinic**  **retention** | 0-1 | 118720 | 61966 | 0.48 | 0.48-0.48 |
|  | 1-2 | 56754 | 10100 | 0.39 | 0.39-0.40 |
|  | 2-3 | 46654 | 7677 | 0.33 | 0.33-0.33 |
|  | 3-4 | 38977 | 7452 | 0.27 | 0.26-0.27 |
|  | 4-5 | 31525 | 7234 | 0.20 | 0.20-0.21 |
|  | 5-6 | 24291 | 6110 | 0.15 | 0.15-0.16 |
