# Supplementary material for: Estimating retention in HIV care accounting for patient transfers: A national laboratory cohort study in South Africa
Source: PLoS Med. 2018 Jun 11;15(6):e1002589. doi: 10.1371/journal.pmed.1002589 (PMC5995345; doi:10.1371/journal.pmed.1002589)
Supplement: S5 Appendix — (DOCX) [file pmed.1002589.s006.docx]

**S5 Appendix. Effect of patient transfer on retention estimates overall in South Africa from ART initiation among 33,201 patients initiating ART from 2004-2006 with a CD4 count < 200 cells/mm^3^ and attrition defined as retained in care on December 31, 2012**

|  | *Interval*  *(years)* | *Beginning*  *N* | *N*  *Attrition* | *Retained* | *95% Confidence*  *Interval* |
| --- | --- | --- | --- | --- | --- |
| **National retention** | 0-1 | 33201 | 2748 | 0.92 | 0.91-0.92 |
|  | 1-2 | 30453 | 2339 | 0.85 | 0.84-0.85 |
|  | 2-3 | 28114 | 1794 | 0.79 | 0.79-0.80 |
|  | 3-4 | 26320 | 1677 | 0.74 | 0.74-0.75 |
|  | 4-5 | 24643 | 1555 | 0.70 | 0.69-0.70 |
|  | 5-6 | 23088 | 1814 | 0.59 | 0.64-0.65 |
| **Clinic**  **retention** | 0-1 | 33201 | 4750 | 0.86 | 0.85-0.86 |
|  | 1-2 | 28451 | 4666 | 0.71 | 0.71-0.72 |
|  | 2-3 | 23785 | 3737 | 0.60 | 0.60-0.61 |
|  | 3-4 | 20048 | 3726 | 0.49 | 0.49-0.50 |
|  | 4-5 | 16322 | 3745 | 0.38 | 0.37-0.38 |
|  | 5-6 | 12577 | 3154 | 0.28 | 0.27-0.29 |
